# Recent Advances in Bunyavirus Glycoprotein Research: Precursor Processing, Receptor Binding and Structure

**DOI:** 10.3390/v13020353

**Published:** 2021-02-23

**Authors:** Ruben J. G. Hulswit, Guido C. Paesen, Thomas A. Bowden, Xiaohong Shi

**Affiliations:** 1 Division of Structural Biology, Wellcome Centre for Human Genetics, University of Oxford, Oxford OX3 7BN, UK; ruben.hulswit@strubi.ox.ac.uk (R.J.G.H.); guido@strubi.ox.ac.uk (G.C.P.); 2 MRC-University of Glasgow Centre for Virus Research, Institute of Infection, Immunity and Inflammation, University of Glasgow, Glasgow G61 1QH, UK

**Keywords:** *Bunyavirales*, bunyavirus, glycoproteins, glycoprotein precursor processing, genomic coding strategy, receptor binding, protein structure

## Abstract

The *Bunyavirales* order accommodates related viruses (bunyaviruses) with segmented, linear, single-stranded, negative- or ambi-sense RNA genomes. Their glycoproteins form capsomeric projections or spikes on the virion surface and play a crucial role in virus entry, assembly, morphogenesis. Bunyavirus glycoproteins are encoded by a single RNA segment as a polyprotein precursor that is co- and post-translationally cleaved by host cell enzymes to yield two mature glycoproteins, Gn and Gc (or GP1 and GP2 in arenaviruses). These glycoproteins undergo extensive N-linked glycosylation and despite their cleavage, remain associated to the virion to form an integral transmembrane glycoprotein complex. This review summarizes recent advances in our understanding of the molecular biology of bunyavirus glycoproteins, including their processing, structure, and known interactions with host factors that facilitate cell entry.

## 1. Introduction

**Figure 1:**
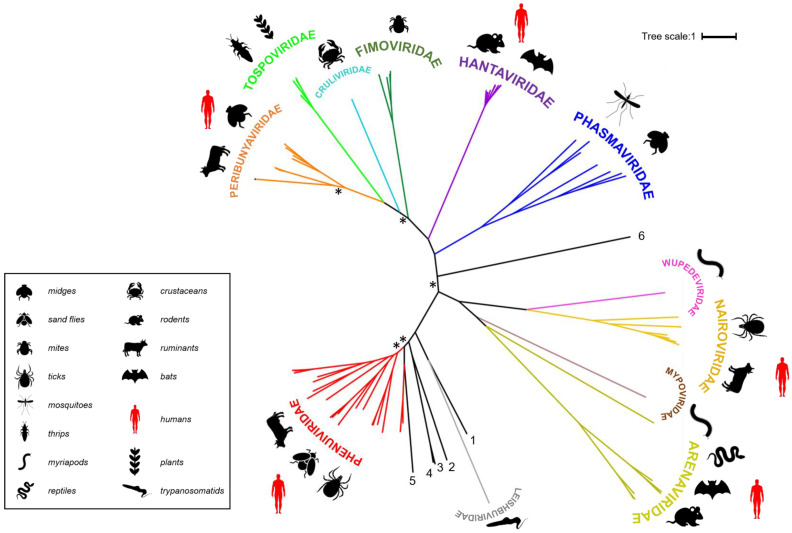
Families belonging to the *Bunyavirales* order. Polymerase sequences of a representative set of bunyaviruses were aligned using MAFFT (Multiple Alignment using Fast Fourier Transform) [20], the alignment was trimmed using trimAI [21] and a phylogenetic tree created in PhyML [22] using the Smart Model Selection option [23]. The unrooted tree was displayed using iTOL (interactive Tree of Life) [24]. Asterisks indicate main branches for which bootstrap support was <0.7. The dataset used for the phylogeny includes all currently assigned bunyavirus families [1], the grouping of which is broadly in line with that presented in a recently published tree [25]. The numbered, black lines in the figure denote bunyavirus species currently not assigned to a family [Wuhan spider virus (**1**), Laurel lake virus (**2**), Hubei blood fluke virus 2 (**3**), Hubei blood fluke virus 1 (**4**), Mothra virus (**5**), Shistocephalus solidus bunya-like virus (**6**)]. Common organisms from which the viruses were isolated are indicated by symbols and viruses known to be capable of zoonotic transmission to humans are annotated with red symbols (the panel at the right denotes what the symbols represent).

Bunyaviruses constitute an expanding and extremely diverse group of RNA viruses with linear, segmented, single-stranded, negative-sense or ambisense RNA genomes, even more so since they were recently re-categorized by the International Committee on Taxonomy of Viruses (ICTV) from a family (*Bunyaviridae*) to an order (*Bunyavirales*) [1]. The order accommodates more than 480 named species (collectively known as bunyaviruses), and is now classified into 12 families: *Arenaviridae*, *Cruliviridae*, *Fimoviridae*, *Hantaviridae*, *Leishbuviridae*, *Mypoviridae*, *Nairoviridae*, *Peribunyaviridae*, *Phasmaviridae*, *Phenuiviridae*, *Tospoviridae* and *Wupedeviridae* [1,2] as illustrated by the polymerase-based phylogenetic tree of the representative members of the order (Figure 1). In line with their potential to unpredictably emerge and cause severe disease, several viruses in the order are now recognized as priority pathogens by the World Health Organization (WHO) [3].

The majority of the bunyaviruses are of little renown or consequence beyond their natural arthropod and mammalian host reservoirs. However, a significant number have repeatedly shown the capacity to cross the species barrier and impose a burden upon human health, animal husbandry and agriculture. Renowned examples include Lassa virus (LASV) (belonging to the *Arenaviridae* family), La Crosse virus (LACV), Schmallenberg virus (SBV) and Oropouche virus (OROV) (all members of the *Peribunyaviridae* family), Hantaan virus (HTNV), Andes virus (ANDV) and Sin Nombre virus (SNV) (*Hantaviridae*), Rift Valley fever virus (RVFV) and severe fever with thrombocytopenia syndrome virus (SFTSV) (*Phenuiviridae*), Crimean-Congo hemorrhagic fever virus (CCHFV) (*Nairoviridae*) [4,5,6]. Tomato spotted wilt virus (TSWV) (*Tospoviridae*) and rice stripe virus (RSV) (*Phenuiviridae*) infect hundreds of plant species, causing great economic losses worldwide [7]. The emergence of novel bunyaviruses and the increasing frequency of outbreaks constitute a growing threat to public health [8,9,10]. Most known bunyaviruses of importance to human health, animal husbandry and agriculture are arthropod-borne (arbo-bunyaviruses). Orthobunyaviruses, phleboviruses and nairoviruses are transmitted by haematophagous arthropods, such as mosquitoes, sandflies and ticks, tospoviruses are transmitted by thrips, and tenuiviruses by planthoppers [5,11,12,13,14,15,16]. Notable exceptions are the arenaviruses and hantaviruses, which use rodents as reservoir hosts. Subsequent human-to-human infections have been reported, including LASV, CCHFV (reviewed in [17]) and ANDV [18,19].

Except for the non-enveloped plant-infecting tenuiviruses, bunyaviruses contain a lipid bilayer envelope that is imbedded with viral glycoprotein spikes and encloses a segmented, single-stranded RNA genome of negative- or ambi-sense polarity. Bunyaviruses replicate in the cytoplasm and assemble on membranes of the Golgi complex from which they bud. Most bunyaviruses contain a tripartite genome, consisting of a large (L), medium (M) and small (S) RNA segment. The L segment encodes the L protein, which has the RNA-dependent RNA polymerase (RdRp) and endonuclease functions, the M segment the glycoprotein precursor (GPC), and the S segment the nucleocapsid protein (N), which encapsidates the genomic RNA. In addition, some bunyaviruses encode nonstructural (NS) proteins, such as NSm (whose gene resides on the M segment) and NSs (on the S segment) [5,11]. In contrast to other bunyavirus families, most arenaviruses contain a bi-segmented RNA genome (L and S), with both segments containing genes in an ambisense orientation. The arenavirus L segment encodes a small RING finger protein (Z) in the genomic sense (gRNA) and an L protein in the antigenomic sense (cRNA), whilst the S segment similarly encodes the GPC (sense) and N protein (antisense) [26]. Tenuiviruses (of the *Phenuiviridae* family) possess unique features that set them apart from other bunyaviruses, such as (i) their existence as filamentous or circular ribonucleoprotein (RNP) particles that lacks a lipid bilayer envelope, (ii) the presence of viral glycoproteins in the infectious (RNP) particles associated with virus infected-plant cells, and (iii) four to six RNA segments with the capacity to encode eight or nine proteins [16,27,28].

The glycoproteins on the surface of virions are of special interest, given their crucial roles in virus assembly and host cell invasion, and the fact that they constitute a tropism restriction factor. Furthermore, bunyavirus envelope glycoproteins are also the primary focus of host neutralizing antibody responses following infection or immunization, making them key targets for vaccine development. This review will report recent advances in bunyavirus glycoprotein research, covering genomic diversity of the GPC-encoding RNA segments, precursor cleavage, glycoprotein architecture and structure, and recognition of host cell receptors. 

## 2. Genomic Organization and Coding Strategy of RNA Segments for Viral Glycoprotein Precursors

**Figure 2:**
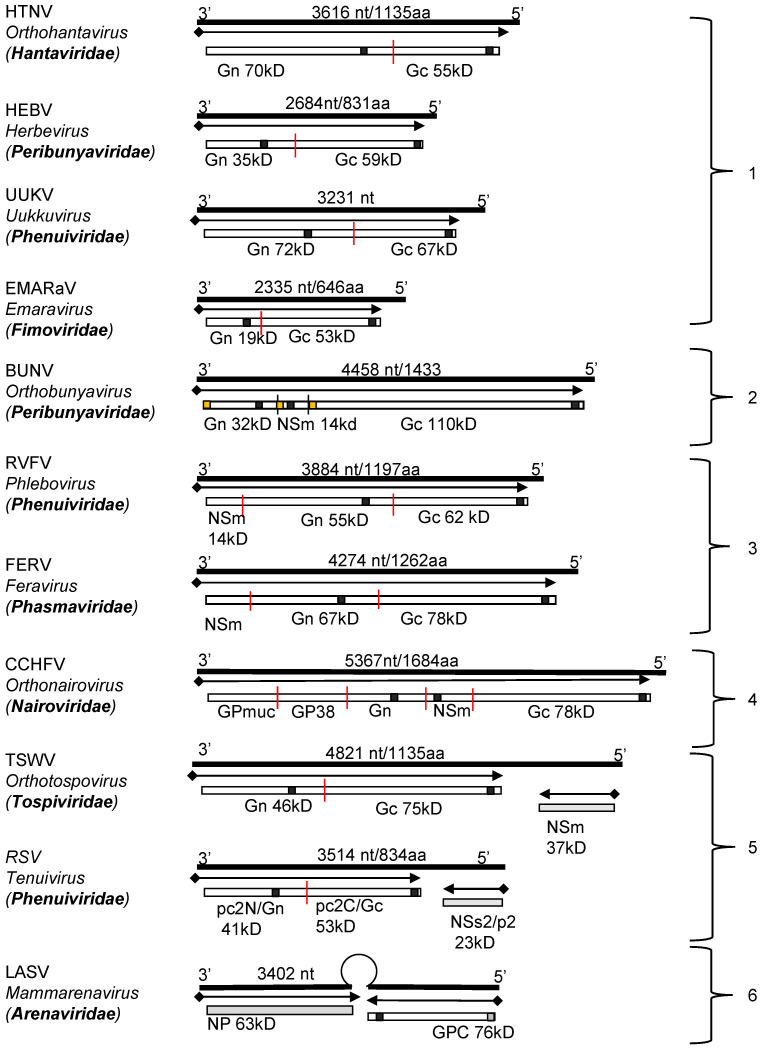
Genomic structure and coding strategies for the viral genomic RNA segments coding for viral glycoprotein precursors (GPCs) from well-studied families within the *Bunyavirales* order. Genomic RNAs (3’ to 5’) are represented by black lines (the numbers of nucleotides and the amino acid of GPC are given above). mRNAs are shown as arrows (♦ indicates host-derived primer sequence at 5’ end by cap-snatching mechanism). Gene products are presented by bars with their approximate size shown underneath. The sites for cleavage are represented by “**|**”. Virus abbreviations: HTNV, Hantaan virus; HEBV, Herbet virus; UUKV, Uukuniemi virus; EMARaV, European mountain ash ringspot-associated virus; BUNV, Bunyamwera virus; RVFV, Rift Valley fever virus; FERV, Fermo virus; CCHFV, Crimean-Congo haemorrhagic fever virus; TSWV, Tomato spotted wilt virus; RSV, rice stripe virus; LASV, Lassa virus. (1) The M segments of hantaviruses (*Hantaviridae*) [33], herbeviruses (*Peribunyaviridae*) [34], Uukuviruses (e.g., UUKV) (*Phenuiviridae*) [35], and the RNA2 segment of the emaraviruses (*Fimoviridae*) [36] encode GPCs containing two structural glycoproteins, Gn and Gc. (2) The M segments of orthobunyaviruses (*Peribunyaviridae*) [11] encode three proteins, with an NSm located between Gn and Gc in the precursor protein. (3) The M segments of phleboviruses (e.g., RVFV) (*Phenuiviridae*) [37] and orthophasmaviruses (e.g., Ferak virus [FRKV] and jonchet virus, [JONV] (*Phasmaviridae*) encode GPCs containing three proteins: Gn and Gc, and an N-terminal NSm [38,39]. (4) The M segment of nairoviruses (e.g., CCHFV) (*Nairoviridae*) encodes a GPC with five proteins: Gn and Gc, and three non-structural proteins; Mucin like protein/domain (MLD), GP38, and NSm [40,41]. The M segments of other members of the family encode precursors for two to four proteins whose exact nature has yet to be confirmed experimentally [42]. (5) The ambisense M segments of orthotospoviruses (*Tospoviridae*) [29] and RNA2 segments of tenuiviruses (*Phenuiviridae*) encode GPCs containing Gn and Gc in the antigenomic sense, and an NSm in the genomic sense [43]. (6) The ambisense S segments of members of the *Hartmanivirus*, *Mammarenavirus* and *Reptarenavirus* genera (*Arenaviridae* family) encode a so-called stable signal peptide (SSP) and the glycoproteins GP1 and GP2 in the genomic sense, and a nucleocapsid protein in the antigenomic sense [26]. Similarly, the M segment of Wēnlǐng frogfish arenaviruses (*Antennavirus* genus) encode their GPCs in the genomic sense and an unknown protein in the anti-genomic sense [44].

The glycoproteins of all viruses in the order *Bunyavirales* are encoded as a GPC within a single open reading frame (ORF). The coding strategies and sizes of products vary considerably from family to family, with the coding-region size ranging from ~2300 nucleotides (Emaraviruses of the *Fimoviridae* family) to ~5500 nucleotides (jonchet jonvirus (JONV) of the *Phasmaviridae* family) (Figure 2). For viruses belonging to the *Peribunyaviridae*, *Nairoviridae* and *Phasmaviridae* families, and for some phleboviruses (*Phenuiviridae*), the M segment also encodes a nonstructural protein (NS), termed NSm, within the glycoprotein precursor. The M segments of orthotospoviruses (*Tospoviridae*), and segment 2 (RNA2) of tenuiviruses (*Phenuiviridae*) follow an ambisense strategy to encode their GPCs and non-structural proteins [29,30] (Figure 2). As mentioned above, arenavirus S segments encode their GPC in the genomic sense and the N protein in the antigenomic sense, the two ORFs being separated by a non-coding, intergenic region containing a hairpin loop involved in transcription termination (reviewed by [31,32]).

The genomic organization of the RNA segments encoding viral glycoprotein precursors of well-studied bunyaviruses can be grouped according to the coding strategies and the gene order of the proteins (Figure 2).

## 3. Processing of Bunyavirus Glycoprotein Precursors

Bunyavirus GPCs are co- and post-translationally cleaved into two mature glycoproteins by a number of specific host cell proteases. The precursors studied so far contain N-terminal and internal signal peptides (SP), except for the arenaviruses, which have a single signal sequence (the above-mentioned SSP) at the N-terminus of the GP1 [45,46,47,48,49]. The SPs are essential for endoplasmic reticulum (ER) membrane translocation and facilitate protein cleavage and maturation. Binding of the nascent precursor SP by signal recognition particles guides the ribosome towards the ER, where the SP becomes inserted into the membrane, directing the remainder of the polypeptide into the ER lumen as it emerges from the ribosome. The SP is co-translationally cleaved from the polypeptide chain by cellular signal peptidases (SPase) [48]. In addition to SPase, several other host proteases that target specific peptide motifs, including signal peptide peptidase (SPP) [50], subtilisin kexin/isozyme-1 (SKI-1/S1P) and furin-like proteases have been shown to process bunyavirus GPCs [40,41,46,51,52].

**Figure 3:**
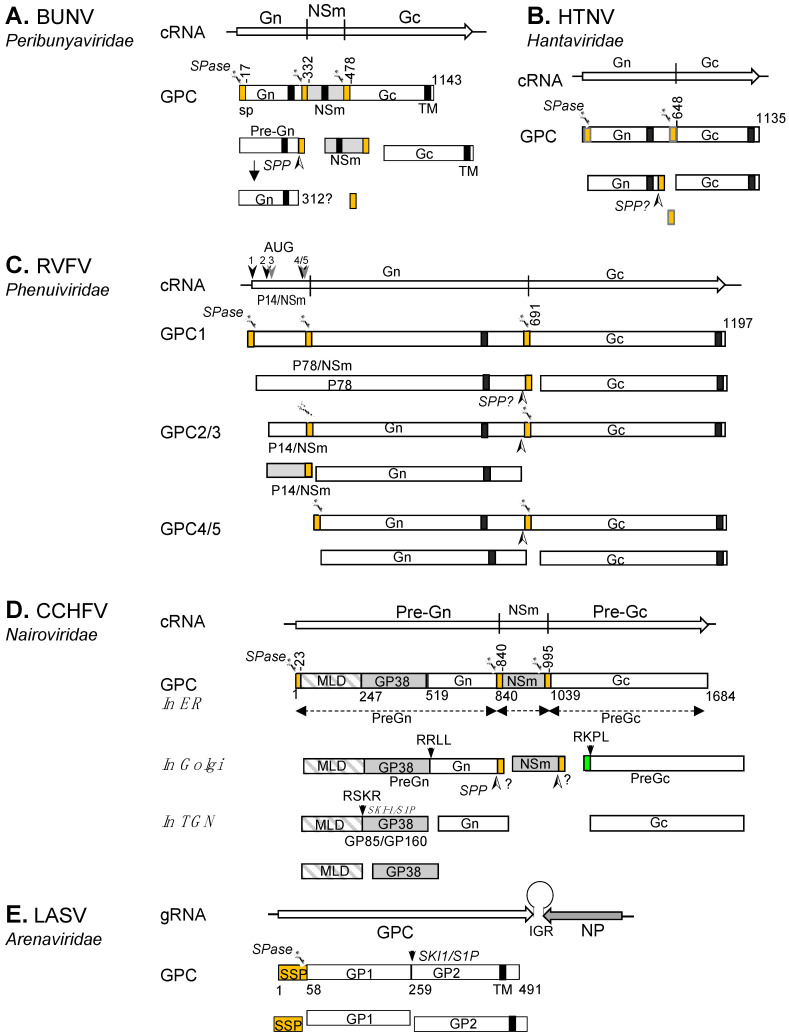
Current models of bunyaviral GPC processing for selected peribunyaviruses, hantaviruses, phenuiviruses, nairoviruses, and arenaviruses. GPC polyproteins are shown for BUNV (**A**), HTNV (**B**), RVFV (**C**), CCHFV (**D**) and LASV (**E**). The signal peptide (SP) and stable SP (SPP, for LASV) are represented in orange. Transmembrane domains (TM) are shown as black boxes. The cleavage sites for signal peptidase (SPase) and signal peptide peptidase (SPP) are marked by scissors and an arrowhead (➣), respectively. The SKI-1/S1P cleavage site is marked by a downward arrow. Nonstructural proteins (NSm and GP38) are shown as grey boxes and the CCHFV mucin domain as a grey and white box. The pre-Gc N-terminal 41 residues that are processed at the RKPR/RKPL motif by a SKI-1/S1P-like protease are shown as green box.

In line with the extensive genetic diversity within the *Bunyavirales* order, varied enzymatic pathways are followed for GPC processing. In the following section, we discuss these pathways for representative viruses of the *Hantaviridae*, *Peribunyaviridae*, *Phenuiviridae*, *Nairoviridae* and *Arenaviridae* families (Figure 3). Although GPC processing has been described for only a few viruses, we believe that in many cases, the mechanisms are shared amongst many members of the same genus or family. 

### 3.1. Orthobunyaviruses (Family Peribunyaviridae)

Bunyamwera virus (BUNV) is the prototype member for both the *Orthobunyavirus* genus and the *Peribunyaviridae* family. The Gn/NSm/Gc coding pattern of BUNV GPC is shared by the viruses of three genera (*Orthobunyavirus*, *Pacuvirus* and *Shangavirus*) in the family with the exception of arthropod-specific herbeviruses (Genus *Herbevirus*), which do not encode NSm [34,53]. We recently described the processing of BUNV GPC by host SPase and signal peptide peptidase (SPP) to generate Gn, Gc and NSm (Figure 3A) [46]. BUNV GPC (Gn/NSm/Gc) contains three SPs that precede the N-termini of each mature product. Co-translational SPase cleavage at the SP cleavage sites in the ER lumen generates three products: a pre-Gn, mature NSm and Gc. Upon SPase cleavage of NSm SP (SP^NSm^; previously known as domain I of NSm [54]), the SP^NSm^ remains linked to the C-terminus of the pre-Gn until the SP is further released by SPP to generate mature Gn. Interestingly, the SP^Gc^ (previously known as domain V of NSm [54]) is not cleaved from the NSm cytoplasmic region (domain IV) and remains an integral part of the protein, rendering the mature NSm domain IV a cytoplasmic loop structure [46]. Whilst the GPC processing details for all other viruses in the family remain to be elucidated, the mechanism of BUNV GPC is likely shared by viruses with a similar coding pattern.

### 3.2. Orthohantaviruses (Family Hantaviridae)

The M segment genomes of viruses within the family *Hantaviridae* encode the GPC, which contains two structural glycoproteins, Gn and Gc [33]. HTNV Gn and Gc have their own SPs at the N-termini and the two proteins are co-translationally cleaved at a conserved ‘WAASA’ pentapeptide motif by the cellular SPase in the ER lumen [47]. Similar to BUNV described above, SPase cleavage leaves SP^Gc^ connected to the C-terminus of Gn (Pre-Gn). Presumably the SP^Gc^ is processed further by SPP or SPP-like proteases (SPPL) during Gn maturation (Figure 3B).

The M segment GPC coding pattern of hantaviruses is shared with those of many bunyaviruses within other bunyaviral families, including herbeviruses (*Peribunyaviridae*), Uukuniemi phlebovirus (UUKV) (*Phenuiviridae*), Emaraviruses (*Fimoviridae*) as well as TSWV (*Tospoviridae*) and RSV tenuivirus (*Phenuiviridae*) (Figure 2). Although yet to be experimentally validated, this suggests that the processing of GPCs in these families resembles that observed for HTNV.

### 3.3. Phleboviruses (Family Phenuiviridae)

The *Phenuiviridae* family contains 19 genera, which include RVFV and UUKV (genus *Uukuvirus*), SFTSV (genus *Bandavirus*) and RSV (genus *Tenuivirus*) [1,2]. The GPC composition and coding strategy in this family is varied (Figure 2). This review will focus on the processing of the RVFV GPC.

RVFV, the type species of the *Phlebovirus* genus, is a human and animal pathogen prevalent in Africa and the Middle East [55,56]. Its M segment encodes a GPC for the non-structural NSm and P78, and the structural Gn and Gc (Figure 3C). The GPC contains three SPs, which are located at the N-termini of P78, Gn, and Gc. Translation initiation takes place at five different AUGs at the N-terminal NSm coding region, producing a nested set of polyprotein precursors, presumably by leaky scanning of the ribosome [37,57,58,59]. The initiation at AUG1 produces a GPC that contains an N-terminal SP (SP^p78^). Cleavage at SP^P78^ and Gc SP (SP^Gc^) by cellular SPase yields the mature Gc and a large P78 glycoprotein, which encompasses NSm and Gn [57,59]. P78 is dispensable for virus replication in tissue culture [60] but was found to be incorporated into mosquito cell-generated RVFV virions [61]. It has therefore been postulated that RVFV P78 is required for virus dissemination in mosquitoes [62]. 

Precursors 2 and 3 (P14/NSm-Gn-Gc) are initiated at the 2^nd^ and 3^rd^ AUGs, respectively, and are processed into three proteins after cleavage at the SP^Gn^ and SP^Gc^ by the cellular SPase: P14/NSm, pre-Gn, and Gc. Upon SPase cleavage, the SP^Gn^ serves as the C-terminal transmembrane domain of P14/NSm (a type II transmembrane domain that targets the protein to the outer membrane of mitochondria, thereby suppressing apoptosis) [63]. Given the cytosolic location of NSm, it is unsurprising that no carbohydrate was observed at the N-glycosylation sequon (N88) on the protein. However, the same site is glycosylated on P78 [37]. Translation initiation at the 4^th^ or 5^th^ AUG produces only Gn and Gc glycoproteins upon SPase cleavage. The precise N-termini of mature Gn and Gc have been determined by amino-acid sequencing of the virion’s Gn and Gc, confirming the predicted SP cleavage sites (at residue Glu154 for Gn and Cys691 for Gc) [57,64]. Presumably, preGns generated from precursors 2/3 (P14/NSm-Gn-Gc) and 4/5 (Gn-Gc) undergo further processing to produce the mature Gn protein by removing the SP^Gc^ from the preGn cytoplasmic tail by SPP or SPPLs, as we described for the maturation of the BUNV Gn protein [46]. 

### 3.4. Tenuiviruses (Family Phenuiviridae)

RSV, the type member of the *Tenuivirus* genus, contains four single strand RNA segments (designated RNA1, 2, 3 and 4) [43]. RNA2 is equivalent to the M segments of other bunyaviruses, has ambisense polarity and encodes a 94 kDa GPC (pc2N-pc2C) in negative sense (cRNA), which is cleaved into two glycoproteins, pc2N and pc2C, corresponding to the Gn and Gc of other bunyaviruses, respectively. A 23 kDa non-structural protein (NSs2) is encoded in the positive sense (gRNA) (Figure 2) [30,65]. 

### 3.5. Orthonairoviruses (Family Nairoviridae)

Despite the diversity of nairovirus M segments, which encode a precursor for two to five proteins, the potential furin-like and SKI-1/S1P-like substrate sites separating individual proteins are conserved [66], suggesting a common processing mechanism, similar to that known for CCHFV. The CCHFV GPC consists of two envelope glycoproteins (Gn and Gc) and three non-structural proteins (MLD, GP38 and NSm) with SPs present at the N-termini of preGn (MLD/GP38/Gn), NSm and Gc (Figure 3D) [67,68]. Co-translational SPase cleavage at these SPs generate PreGn (MLD/GP38/Gn), NSm and preGc. In the Golgi complex, PreGn undergoes cleavage at the motif RRLL by SKI-1/S1P to separate the N-terminal MLD-GP38 from mature Gn [40,41,51,68,69]. The MLD-GP38 is further cleaved at the RSKR motif by a furin-like proprotein convertase to yield MLD and GP38. MLD and GP38, as well as the uncleaved form (GP85/GP160), are secreted from the CCHFV-infected cells [41]. Using a CCHFV virus-like particle (VLP) approach, it was demonstrated that MLD-GP38 (GP85/GP160) is required for Golgi targeting and maturation of Gc [70]. In the Golgi, Gc matures through the removal of 41 residues from the N-terminus of preGc by an SKI-1/S1P-like protease at the RKPR/RKPL motif [40,51]. Presumably, an SPP or SPPL removes the SP^NSm^ from the Gn cytoplasmic tail in the ER or Golgi. 

### 3.6. Arenaviruses (Family Arenaviridae)

The family *Arenaviridae* was recently placed within the *Bunyavirales* order [71]. In *Hartmanivirus*, *Mammarenavirus* and *Reptarenavirus* genera the GPCs are encoded in the genomic sense by the S segment of their bi-segmented genomes, whilst in the *Antennavirus* genus they are encoded by the M segment of their tri-segmented genomes [26]. Unlike other bunyaviruses, the arenavirus glycoprotein precursor contains only a single stable SP (SSP), which is located at the N-terminus (Figure 3E). During glycoprotein maturation, the SSP is cleaved from the precursor by a cellular SPase, and GP1 is separated from GP2 by subtilase SK1/S1P [52,72,73,74,75]. Unique to arenaviruses, the SSP remains stable upon cleavage by cellular SPase in the ER and associates with GP1/GP2 heterodimers to form an SSP/GP1/GP2 complex [76,77]. The SSP is myristoylated and rearranges to translocate its C-terminus back to the cytosolic side of the membrane, rendering it a double membrane-spanning protein with both the N- and C-termini residing in the cytosol [78]. The SSP is essential for viral infectivity and plays a crucial role in processing and maturation of the precursor, intracellular trafficking of GP1-GP2 to the Golgi, and pH-dependent membrane fusion activity [79,80,81]. 

## 4. Receptors for Bunyavirus Entry

For many enveloped viruses, entry is initiated by the interaction of their envelope glycoproteins with host cell surface receptor(s) [82,83,84,85]. Reflective of the broad range of mammalian, invertebrate, and plant species known to function as virus reservoirs, bunyaviruses utilize a wide variety of cell receptors/co-receptors to achieve this process. While the receptors for the majority of bunyaviruses are still unknown, many cellular receptors and host cofactors have been identified (Table 1). Some excellent reviews on receptors and co-receptors and other cellular factors for the entry of bunyaviruses were recently published [10,86,87,88]. In Table 1 we list receptors and other cellular factors that have been identified so far to play a role in cell entry of bunyaviruses.

While the enveloped Bunyaviruses enter cells via receptor-mediated endocytosis, many details of their endocytic pathways remain uncharacterized [10]. The wide diversity of the virus species, vectors, hosts and receptors for entry implies that bunyaviruses exploit more than one host endocytic route (Table 2). Indeed, an increasing number of studies have shown that bunyaviruses are transported into the low pH endosomal lumen via different endocytosis pathways. Fusion of the virion and endosome, which is triggered following exposure of the virion to low pH environment, has been shown to trigger conformational changes to the glycoproteins for many bunyaviruses (see Section 5 structure of bunyavirus envelope glycoproteins for detail). Such a process allows delivery of the viral ribonucleoprotein (RNP) into the cytosol to initiate viral replication. A notable exception is the infection of plant cells by plant-infecting bunyaviruses, where viral RNP is delivered into the cytoplasm by vectors that are able to breach the structural barrier of plant cells [89]. Several comprehensive reviews of bunyaviruses entry were recently published by Albornoz [10], Leger and Lozach [14], Chen et al. [89], Hallam [90] and Mittler et al. [91]. For viruses within the *Phenuiviridae,* please see an in-depth review of receptors and entry by Koch et al. [88].

**Table 1:** Host cell receptors/co-receptors for entry of viruses in the *Bunyavirales* order.

Genus/Family	Receptor/Co-Receptors	Virus Name	References
*Mammarenavirus* *Arenaviridae*	α-DG	LASV, LCMV, OLVV, MOBV, LATV	[92,93]
	LAMP1	LASV	[94,95]
	TfR1 (CD71)	MACV, JUNV, WWAV, GTOV, SBAV, CHPV, TAMV	[96,97,98,99]
	DC-SIGN, LSECtin	LASV, LCMV	[100,101]
	Axl, Tyro3	LASV, LCMV	[100,101] [102]
	NRP2	LUJV	[103]
	Tetraspanin (CD63)		
	(VGCCs)	JUNV	[104]
*Orthobunyavirus* *Peribunyaviridae*	HSPG	AKAV, SBV	[105]
	DC-SIGN	GERV, LACV	[106,107]
*Orthohantavirus* *Hantaviridae*	αvβ_3_ integrins	SNV, NYV, HTNV, SEOV, PUUV, ANDV	[108,109,110]
	PCDH1	ANDV, SNV, PHV, MAPV	[111]
	β2 integrin	HTNV	[112]
	α5β1 integrin	PHV	[108]
	DAF (CD55)	HTNV, SNV	[113,114]
	gC1qR	HTNV	[115]
	70-kDa protein	HTNV	[116]
*Orthonairovirus* *Nairoviridae*	DC-SIGN	CCHFV	[117]
	Nucleolin		[118]
*Phlebovirus* *Phenuiviridae*	DC-SIGN	RVFV, TOSV, PTV	[106,119,120]
	L-SIGN (CD209L)	RVFV, TOSV	[119]
	HSPG	RVFV, TOSV,	[121,122,123]
	PNASEK	RVFV	[124]
*Uukuvirus* *Phenuiviridae*	DC-SIGN L-SIGN (CD209L)	UUKV	[106,119]
*Bandavirus* *Phenuiviridae*	DC-SIGN, DC-SIGNR, LSECtin	SFTSV	[107,120]
	NMMHC-IIA		[125,126]
*Tenuivirus* *Phenuiviridae*	LsTUB	RSV	[127]
*Orthotospovirus* *Tospoviridae*	50 kDa thrips protein	TSWV	[128,129]
	94 kDa thrips protein		[130]
	TSWV Gn-interacting thrips proteins		[131]

AKAV, Akabane virus; α-DG, alpha-dystroglycan; ANDV, Andes virus; Axl, Axl receptor tyrosine kinase; CCHFV, Crimean-Congo hemorrhagic fever virus; CHPV, Chapare virus; DAF, Decay-accelerating factor; DC-SIGN, Dendritic cell-specific intracellular adhesion molecule-3-grabbing non integrin; DC-SIGNR, DC-SIGN-related; GERV, Germiston virus; GTOV, Guanarito virus; HSPG, Heparan sulfate proteoglycan; HTNV, Hantaan virus; JUNV, Junín virus; LACV, La Crosse virus; LAMP1, Lysosome-associated membrane protein 1; LASV, Lassa virus; LATV, Latino virus; LCMV, lymphocytic choriomeningitis virus; LSECtin, C-type lectin receptor; L-SIGN, liver-specific intercellular adhesion molecule-3-grabbing non-integrin; LsTUB; *L. striatellus* α- tubulin 2; LUJV, Lujo virus; MACV, Machupo virus; MAPV, Maporal virus; MOBV, Mobala virus; NRP2, Neuropilin-2; NYV, New York virus; NMMHC-IIA, Non-muscle myosin heavy chain IIA; OLVV, Oliveros virus; PCDH1, Protocadherin-1; PHV, Prospect Hill virus, PNASEK, Proteinase K; PTV, Punta Toro virus; PUUV, Puumala virus; RSV, rice stripe virus; RVFV, Rift Valley fever virus; SBAV, Sabiá virus; SBV, Schmallenberg virus; SEOV, Seoul virus; SFTSV, severe fever with thrombocytopenia syndrome virus; SNV, Sin Nombre virus; TAMV, Tamiami virus; TfR1, Transferrin receptor protein 1; TOSV, Toscana virus; TSWV, Tomato spotted wilt virus; Tyro3, tyrosine-protein kinase receptor 3; UUK, Uukuniemi virus; VGCCs, Voltage-gated calcium channels; WWAV, Whitewater Arroyo virus.

## 5. Structure of Bunyavirus Envelope Glycoproteins

**Figure 4:**
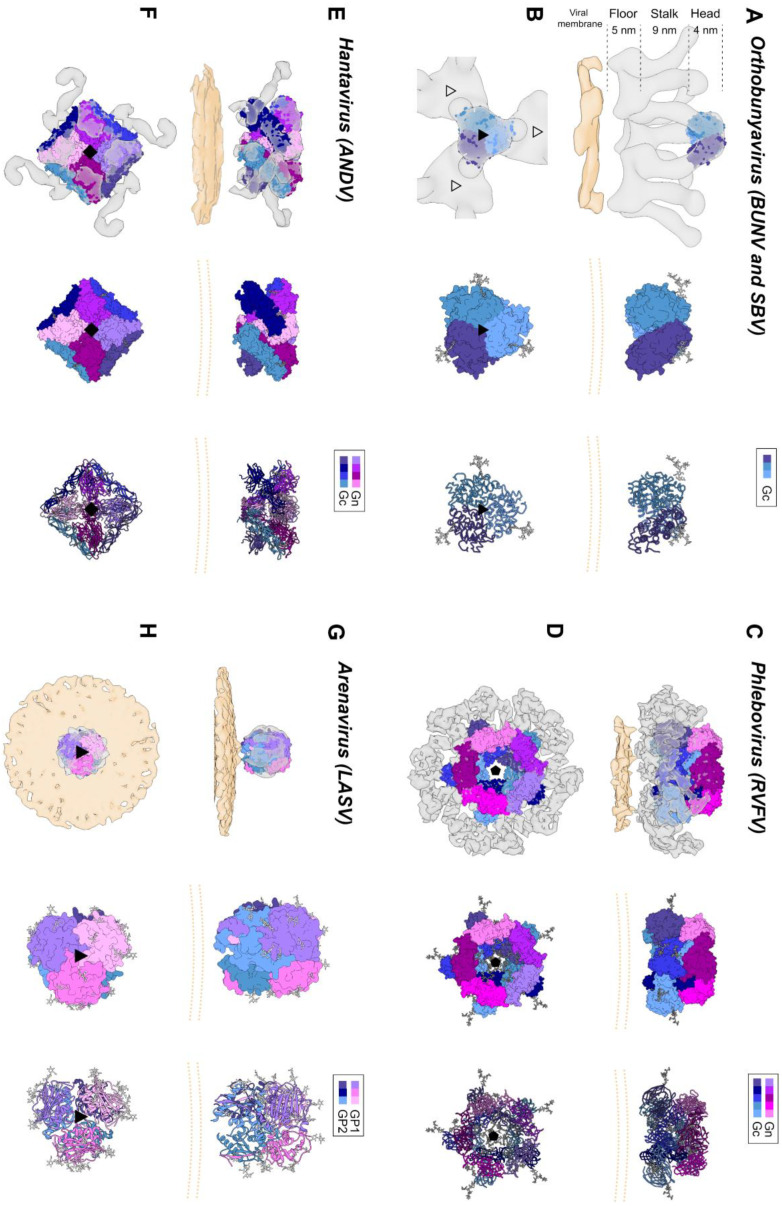
Envelope glycoprotein ultrastructure of orthobunya-, hanta-, phlebo- and arenavirus particles. (**A**) *Left panel*: an EM reconstruction of an orthobunyaviral envelope glycoprotein (in grey; EMD-2352^7^) obtained using Bunyamwera (BUNV) particles, to which the trimeric BUNV Gc head domain structure (PDB: 6H3V^8^) was fitted, in side view. The center of the tripodal organization is linked together by the membrane-distal, N-terminal extension of the Gc. The different regions of the Gn-Gc spike complex (head, stalk, floor) are indicated on the left with corresponding heights. The head and stalk regions make up the N-terminal extension of the peribunyaviral Gc glycoprotein, whereas the floor region is thought to represent the Gn ectodomain and the fusogenic C-terminal region of the Gc ectodomain. *Middle panel*: zoomed-in surface representation of the Gc head domain trimer. Each of the protomers is colored in a different shade of blue. *Right panel*: Ribbon presentation of the middle panel. (**B**) Top views of the three upper panels. *Left panel*: the different threefold symmetry axes are indicated by open or solid triangles. (**C**) *Left panel*: EM density corresponding to the region of a phleboviral envelope Gn-Gc complex that exhibits five-fold symmetry (in grey; EMD-4201 [151]), obtained using Rift Valley fever virus (RVFV) particles. RVFV Gn and Gc crystal structures were fitted into this this pentameric RVFV envelope glycoprotein capsomer (PDB: 6F9F [151]), in side view. N-terminal regions of the Gn (membrane distal) are indicated in shades of purple and pink and the Gc ectodomain (membrane proximal) in shades of blue. Glycoprotein EM density is shown in grey. *Middle panel*: surface representation of the pentameric organization of Gn/Gc heterodimers. *Right panel*: Ribbon presentation of the middle panel. (**D**) Top views of the three upper panels. *Left panel*: the fivefold symmetry axis is indicated by a solid pentagon. (**E**) *Left panel*: the EM density of a hantaviral envelope Gn-Gc complex (in grey; EMD-11236, [154]) obtained using Tula virus (TULV) particles to which a model of tetrameric (Gn-Gc)_4_ ANDV spike (PDB: 6ZJM, [154]) was fitted, in side view. ANDV Gn is indicated in shades of purple and pink and ANDV Gc in shades of blue. *Middle panel*: surface representation of the tetrameric organization of Gn/Gc heterodimers. *Right panel*: ribbon presentation of the middle panel. (**F**) Top views of the three upper panels. *Left panel*: the fourfold symmetry axis is indicated by a solid square. (**G**) *Left panel*: EM density of a trimeric arenavirus envelope glycoprotein spike (in grey; EMD-3290 [155]) obtained using Lassa virus (LASV) particles to which the trimeric LASV GP1-GP2 envelope glycoprotein (PDB: 5VK2 [156]) was fitted, in side view. GP1 protomers (membrane distal) are indicated in shades of purple and pink and GP2 protomers (membrane proximal) in shades of blue. Glycoprotein EM density is shown in grey. *Middle panel*: zoomed-in surface representation of the trimeric organization of LASV GP1/GP2 heterodimers. *Right panel*: Ribbon presentation of the middle panel. (**H**) Top views of the three upper panels. *Left panel*: the threefold symmetry axis is indicated by a solid triangle. In all structural representations crystallographically observed glycans are shown as white sticks. In the case of the RVFV Gn-Gc pentameric assembly, glycan chains were modelled onto N-linked glycosylation sites. The position of the viral membrane is shown as a yellow surface or two yellow dashed lines. To emphasize the protein components of the reconstructions, lipid bilayer EM densities were rendered at a lower sigma level than the protein surfaces. A color legend is shown on the top right-hand side of each panel.

**Figure 5:**
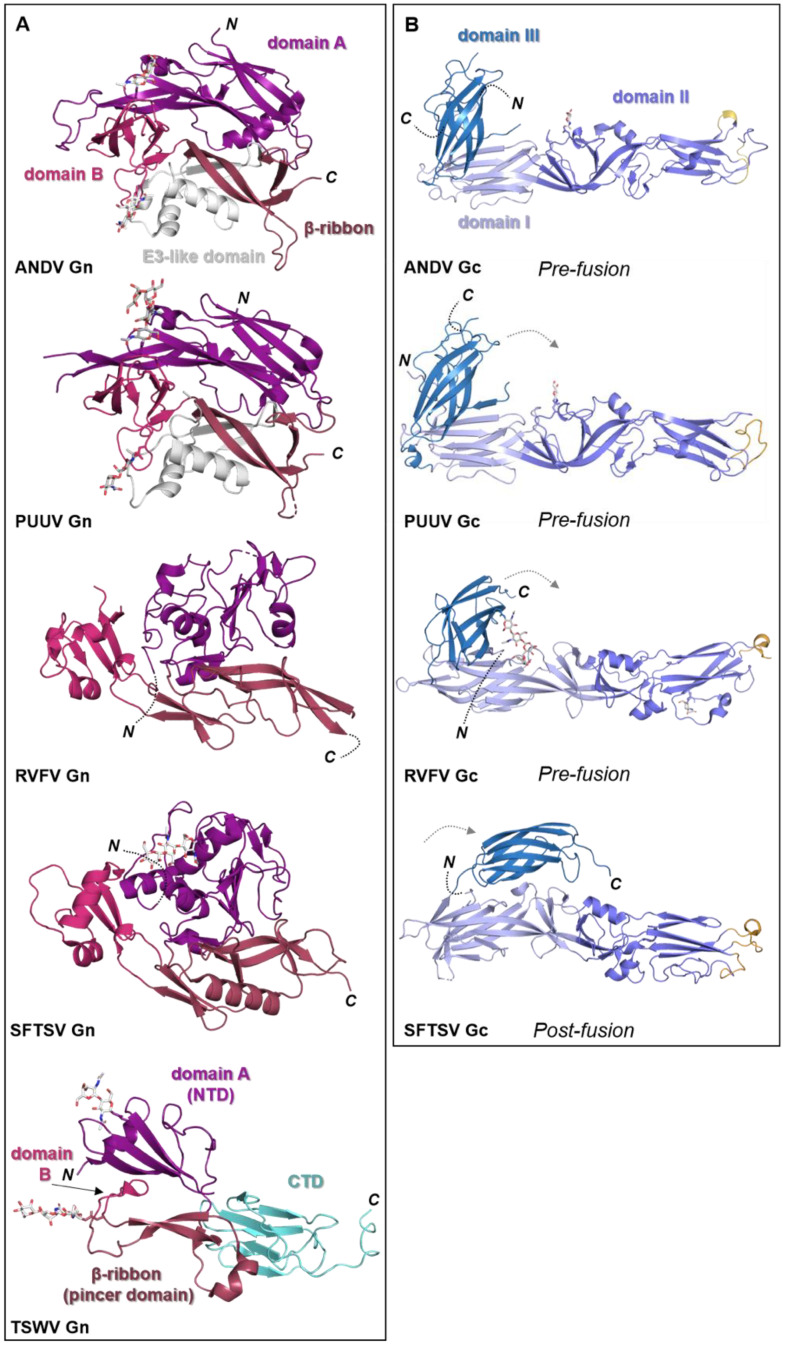
Known structural features of bunyaviral Gn and Gc envelope proteins. (**A**) The Gn envelope glycoprotein displays limited structural similarity across bunyavirus families. Five crystal structures of Gn ectodomain regions of different bunyaviruses are shown. *Upper panel*: cartoon representation of the Gn N-terminal region of the ectodomain of the New World orthohantavirus ANDV (PDB: 6Y6P [154]), which displays a four-domain architecture (domain A, deep purple; a β-ribbon domain, purple-brown; domain B, warm pink; and a domain reminiscent of the alphavirus E3 protein, white). *Second panel*: cartoon representation of the Gn N-terminal region of the ectodomain of the Old World orthohantavirus PUUV (PDB: 5FXU [165]). *Third panel*: cartoon representation of the N-terminal region of the Gn ectodomain from RVFV (PDB: 6F8P [151]). *Fourth panel*: cartoon representation of the N-terminal region of the Gn ectodomain from SFTSV (PDB: 5Y10 [166]). Interestingly, SFTSV Gn contains a region reminiscent of the E3-like domain observed in hantavirus Gn proteins. *Bottom panel*: cartoon representation of the Gn ectodomain from TSWV (PDB: 6Y9L [167]). TSWV Gn displays a largely conserved three-domain architecture in which domain B is reduced to a β-hairpin. The C-terminal domain (CTD) comprises a β-sandwich domain (cyan) (please see Figure 6). (**B**) Structurally characterized bunyaviral Gc fusion proteins display a conserved class II fusion protein architecture (domain I, light blue; domain II, slate blue; domain III, sky blue). Four crystal structures of the Gc ectodomain of different bunyaviruses are shown in a putative pre-fusion conformation (except SFTSV Gc for which a post-fusion state was determined). The dashed grey arrow indicates the movement of domain III between putative pre- and post-fusion conformations. Fusion loop(s) are indicated in bright orange. *Top panel*: crystal structure of the ANDV New World orthohantavirus Gc protein ectodomain in its pre-fusion conformation (PDB: 6Y5F [154]). *Second panel*: crystal structure of the Old World orthohantavirus PUUV Gc protein ectodomain in its pre-fusion conformation (PDB: 7B09 [168]). *Third panel*: crystal structure of the RVFV phlebovirus Gc protein ectodomain in its pre-fusion conformation (PDB: 4HJ1 [160]). *Bottom panel*: crystal structure of the SFTSV Gc protein ectodomain in its post-fusion conformation (PDB: 5G47 [169]). Note that the position of domain III has shifted from the tip of domain I in pre-fusion conformations towards the border of domains I and II in this post-fusion state. In all structural representations crystallographically observed glycans are shown as white sticks.

**Figure 6:**
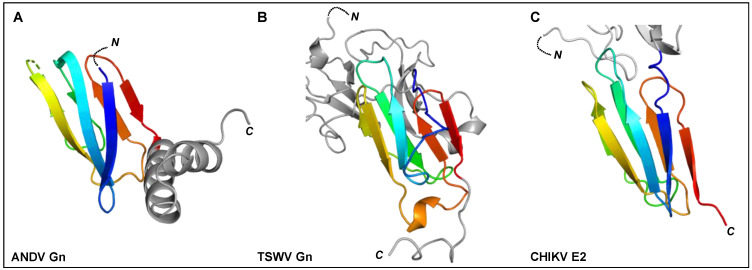
The C-terminal regions of ANDV Gn, TSWV Gn, and alphavirus E2 ectodomains have been shown to contain a seven-stranded β-sandwich fold. Rainbow cartoon representation of the aligned C-terminal β-sandwich domains of ANDV Gn, TSWV Gn and CHIKV E2. (**A**) The N-terminus of the displayed ANDV Gn structure (PDB: 6YRB, [154]) connects to the multi-domain globular region presented in Figure 5 and the C-terminus connects to an α-helical hairpin (grey cartoon), also known as the ‘base’ domain. (**B**) The N-terminus of the displayed TSWV Gn structure (PDB: 6Y9L, [167]) connects to the multi-domain globular region (grey cartoon, also presented in Figure 5) and the C-terminus connects to the transmembrane domain. (**C**) The N-terminus of the displayed CHIKV E2 structure (PDB: 3N43, [170]) connects to a multi-domain globular region (partially shown by grey cartoon) and the C-terminus connects to the transmembrane domain. The N- and C-termini of the β-sandwich folds are colored blue and red, respectively, and the N- and C-termini of the structures are indicated.

**Figure 7:**
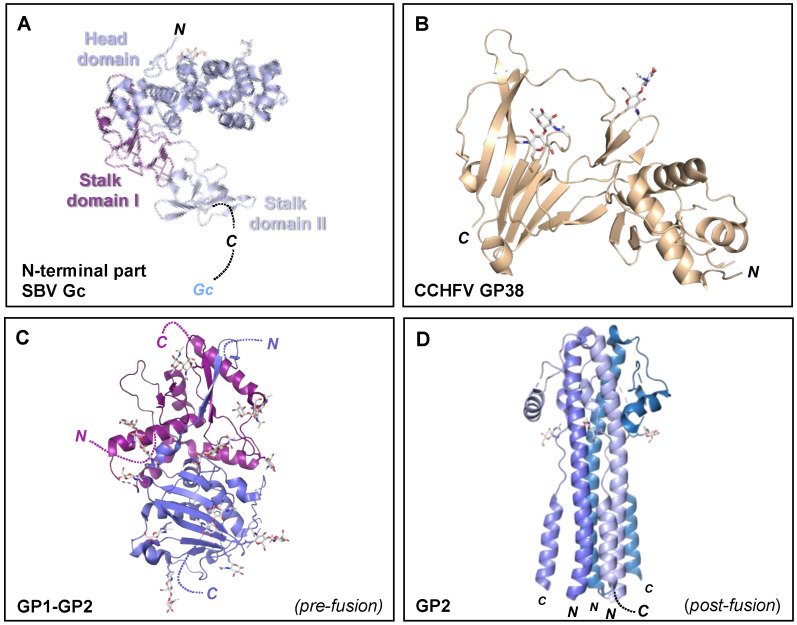
Crystal structures of the orthobunyavirus Gc N-terminal region, CCHFV GP38, and arenavirus glycoproteins. (**A**) Crystal structure of the multi-domain N-terminal region of SBV Gc (head domain, light blue; stalk domain I, violet purple; stalk domain II, blue white) (PDB: 6H3S [153]). The N- terminus of the protein is indicated, as is the C-terminus that connects to the Gc protein. (**B**) Crystal structure of CCHFV GP38 protein (PDB: 6VKF [175]). The N- and C-termini of the protein are indicated. (**C**) Single protomer of the trimeric LASV GP crystal structure (PDB: 5VK2 [156]). The N- and C-termini of the GP1 and GP2 components are indicated. (**D**) Crystal structure of the trimeric post-fusion state of LASV GP2 (PDB: 5OMI [176]). N- and C-termini of the different GP2 protomers are indicated. In all structural representations crystallograhpically observed glycans are shown as white sticks.

**Figure 8:**
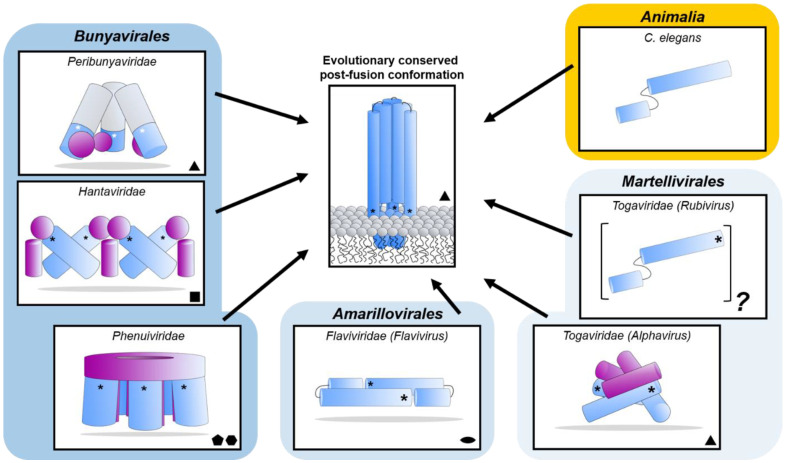
Diverse class II fusion protein architectures converge on an evolutionary conserved trimeric post-fusion conformation. Schematic representation of class II fusion proteins in their pre-fusion oligomeric state. The schematic assemblies are based on crystal structures and/or cryoEM reconstructions (*Peribunyaviridae*: BUNV (EMD-2352 [163]) and SBV (PDB: 6H3V [153]); *Hantaviridae*: TULV (EMD-3364 [165]); *Phenuiviridae*: RVFV (EMD-4201 and PDB: 6F9F [151]); *Flaviviridae*: (PDB: 4UTC [189]); *Togaviridae*: Alphavirus eastern equine encephalitis virus (EEEV; PDB: 6MX4 [190]), Rubivirus RUBV (PDB: 4ADJ[191]); *C. elegans* (PDB: 4OJC [149]). The elongated structures of class II fusion proteins are shown as blue shapes (Gc for members of the *Bunyavirales*, E1 for *Togaviridae*, E for *Flaviviridae* and EFF-1 for the cellular *C. elegans* protein). Putative fusion protein stabilizing entities present on mature viral particles, are shown as purple shapes and have been hypothesized to prevent premature fusion activation (*Bunyavirales*: Gn, *Togaviridae*: E2). The E3 protein has been shown to be present in some alphavirus particles [170] but is omitted from this representation for clarity. The level of symmetry of each of the protein assemblies is indicated by symmetry symbols at the bottom right-hand corner. The approximate position of the fusion loop(s) is indicated with an asterisk (*) for each panel. In the case of peribunyaviruses, the exact location of the fusion loop (white asterisk) within the Gc protein is currently not known, but was inferred from the location of the N-terminal extensions within the tripodal EM reconstruction [153,163] and the C-terminal positioning of Gc transmembrane domains. Note that, although *C. elegans* EFF-1 (epithelial fusion failure 1) protein presents a class II fusogen architecture, it does not contain a fusion loop. Fusion is believed to be initiated by trimerization of the plasma membrane anchored EFF-1 ectodomains protruding in the extracellular space [149]. The grey region of the column shown for *Peribunyaviridae* represents the N-terminal extension of the Gc fusion protein, which has not been observed in other bunyavirus glycoproteins. The pre-fusion oligomeric state of EFF-1 has been observed to be monomeric on the plasma membrane [192]. The pre-fusion oligomeric state of rubella virus E1 on the virus membrane is currently unknown and therefore represented as a protomer of an unknown oligomeric assembly. The fusion proteins of alpha- (e.g., Semliki Forest virus (SFV), chikungunya virus (CHIKV)) and flaviviruses (e.g., dengue virus (DENV), zika virus (ZIKV)) are structurally related despite a lack of detectable sequence conservation and are therefore positioned next to each other in the diagram. Similarly, phenuivirus Gc has been shown to be genetically more closely related to the fusion envelope (E) proteins of flaviviruses than to those of other genera in its own order [152]. These proteins are placed next to each other to represent this predicted relationship. The box depicting the cellular EFF-1 protein is colored in yellow as to oppose the boxes in different shades of blue which all contain viral fusion proteins.

The bunyavirus glycoproteins Gn and Gc (or SSP, GP1 and GP2 in the case of arenaviruses) form spikes on the lipid bilayer envelope of the virion and facilitate viral invasion of a host cell. High resolution structures of bunyavirus glycoproteins in isolation, and low resolution cryoEM studies of entire virions/VLPs, which are often pleomorphic in shape or deviate from icosahedral symmetry [147], have been predominantly limited to orthobunyaviruses, phenuiviruses, hantaviruses, tospoviruses and arenaviruses. Although these glycoproteins assemble and give rise to diverse quaternary architectures (Figure 4), conserved structural features and folds have been observed within the order (Figure 5 and Figure 6), with the notable exception of arenaviruses (please see Section 5.6 and Figure 7). In particular and consistent with predictions [148], the Gc glycoprotein from phleboviruses, bandaviruses, and hantaviruses have been shown to display a class II fusion architecture consisting of three domains, termed I-III, which forms trimers following merger of the virion envelope and target membranes (Figure 8; reviewed in [149,150]). Although structurally diverse, the cognate Gn glycoprotein from these same virus groups shares similarities with the E2 glycoprotein of alphaviruses [151,152]. We also describe how these relatively conserved structural features contrast those of the characterized arenaviruses in Section 5.6.

### 5.1. Orthobunyaviruses (Family Peribunyaviridae)

High resolution structural information is currently limited to the N-terminal region of the orthobunyavirus Gc. The crystal structure of the N-terminal half of SBV Gc revealed an elongated multi-domain assembly composed of an α-helical head domain connected to a stalk region composed of two β-sheet subdomains (Figure 7A) [153]. Additional crystal structures of the α-helical head domains from BUNV, OROV and LACV Gc demonstrated that this region of the glycoprotein is relatively conserved amongst genetically diverse orthobunyaviruses [153].

Interestingly, the N-terminal half of the BUNV Gc ectodomain has been shown to be dispensable for the virus replication cycle *in vitro*, but can serve to modulate the fusogenicity of the protein [157,158]. The structure of the C-terminal half of the orthobunyaviral Gc ectodomain remains unknown, however, it has been proposed to form a pH-dependent class II fusion fold [148,159] akin to those observed in phleboviruses [160] and hantaviruses [161,162]. Similarly, the structure of the orthobunyavirus Gn has yet to be reported. 

Electron cryo-tomography (cryoET) of purified BUNV, the prototypic orthobunyavirus, has demonstrated that the Gn-Gc spike complex projects approximately 18 nm from the viral membrane (Figure 4A,B) [163]. Sub-tomogram averaging of the spike yielded a low resolution (~3-nm) reconstruction and revealed that Gn and Gc assemble to form a pH-sensitive tripod-like arrangement with a locally ordered lattice formed by trimeric surfaces located in both the membrane distal ‘head’ region and the membrane proximal ‘floor’ region (Figure 4A,B) [163,164]. Interestingly, the structure of the N-terminal region of BUNV and La Crosse virus (LACV) Gc was observed to form extensive trimeric contacts in the crystal, and this trimer fits well into the membrane distal pyramidal region of the cryoET-derived reconstruction [153]. These combined observations are consistent with the Gn and the C-terminal region of the orthobunyaviral Gc occupying the membrane proximal region of the orthobunyaviral Gn-Gc complex (Figure 4A,B). Future studies will shed light on the functional interactions between the head and stalk regions with the fusogenic C-terminal region of the orthobunyaviral Gc, and on the functional role of the smaller Gn.

### 5.2. Phenuiviruses (Family Phenuiviridae) 

Crystal structures of N-terminal ectodomain regions from RVFV (genus *Phlebovirus*) and SFTSV (genus *Bandavirus*) Gn proteins have recently been determined, revealing that both glycoproteins form a three-domain architecture composed of a mixed α-helical/β-stranded N-terminal domain, termed herein as ‘domain A’, a ‘β-ribbon domain’, and a ‘domain B’ [151,166,171,172] (Figure 5A). Although RVFV Gn and SFTSV Gn present the same overall fold, comparison reveals relatively large differences (~3 Å root-mean-square deviation), demonstrating that structural variation exists across the family [166].

The structure of the phenuivirus Gc ectodomain has been determined for RVFV, SFTSV, and Heartland virus (HRTV) [160,169,173,174]. In contrast to the phleboviral Gn, the Gc is relatively conserved and displays a class-II fusion fold (Figure 5B). The three Gc domains, termed I-III, are composed predominantly of β-sheets (reviewed in [149,150]). Fusion loops have been identified in domain II, which inserts into the target membrane of the host cell. A conserved cavity nearby the fusion loop of RVFV interacts with glycerophospholipids and contributes to the interaction of Gc with the target membrane [174]. Comparison of putative pre- and post-fusion conformations of RVFV, HRTV, and SFTSV Gc glycoproteins provides a structural basis for understanding the fusogenic rearrangements that these glycoproteins undergo to facilitate merger of the virus and cell membranes following endocytic uptake of the virus.

Initial structural studies of purified RVFV and UUKV by EM revealed that Gn-Gc heterodimers form an icosahedral lattice (*T* = 12) with 122 distinct capsomers (12 pentons and 110 hexons) (Figure 4C,D) [177,178,179,180]. The resolution achieved by these investigations was restricted to approximately 2-nm, a limitation that has been attributed to the flexibility of the Gn-Gc glycoprotein assembly [151]. A more recent set of reconstructions of RVFV by Halldorsson and Li et al. benefited from optimized sample preparation approaches (i.e., virus fixation), improved direct detector technologies and data processing strategies (i.e., a localized reconstruction approach) for EM, and yielded an improved resolution of approximately 1-nm [151]. While the resolution achieved by this study was insufficient to define the specificity of inter-subunit interactions formed by Gn and Gc, it was able to localize the crystallized fragments of the two glycoproteins, where the N-terminal region of the Gn ectodomain is placed at the membrane-distal region of the viral envelope, covering much of the Gc glycoprotein. It has been postulated that placement of the Gn shields the hydrophobic fusion loop of the Gc, preventing premature fusion. Shielding of the Gc is consistent with sera binding data from RVFV infected individuals [181], which demonstrated that antibodies preferentially target the Gn. Further cryoET imaging and reconstructions of acidified RVFV have given clues to the repositioning of the Gn and are consistent with the requirement for Gc to extend towards the target membrane [151]. Future structural studies will benefit from comparison of the higher-order ultrastructure of genetically diverse phenuiviruses, such as SFTSV and HRTV, and to assess whether such assemblies and structural transitions are conserved across the family.

### 5.3. Hantaviruses (Family Hantaviridae)

Crystal structures of fragments from the Gn ectodomains of Maporal virus (MAPV), ANDV, HTNV and PUUV hantaviruses have revealed that the glycoprotein consists of an N-terminal ‘head’, which forms a fold similar to that exhibited by the Gn of phenuiviruses and the E2 of alphaviruses (Figure 5A) [151,152,154,165,182]. The head connects to a β-sandwich fold (Figure 6A) and a C-terminal tetrameric ‘base’ domain [154]. Crystallographic investigations of the cognate Gc from these viruses have revealed that the glycoprotein comprises a class II fusion fold [154,161,162] (Figure 5B). Different from other class II fusion proteins, hantavirus Gc has an N-terminal extension (Gc N-tail), which has been postulated to stabilize the Gc post-fusion trimer. The structure of the Gn cytoplasmic tail (CT) from ANDV was resolved by nuclear magnetic resonance (NMR) and shown to contain conserved dual CCHC-type classical ββα-fold zinc fingers, suggestive that hantavirus Gn CTs are involved in binding the RNP during virion assembly [183,184].

Initial negative-stain EM studies of HTNV revealed a distinct ‘grid-like pattern’ on the virion surface [185]. Interestingly, (Gn-Gc)_4_, which form the spikes that constitute these grids, has been proposed to exist in equilibrium between two conformational states, termed ‘closed’ and ‘open’ [186]. The ‘closed’ form does not bind membranes at neutral pH, likely due to occlusion of Gc-resident fusion loops, but is capable of undergoing fusogenic rearrangements upon exposure to acidic environments. In the ‘open’ form, Gc fusion loops have been proposed to be exposed as they can bind target membranes at neutral pH but are unable to fuse the target and viral membranes. 

The ultrastructure of the putative ‘closed’ form has been studied by cryoET analysis of Tula virus (TULV) and HTNV, which revealed that (Gn-Gc)_4_ form a square-like organization that extends approximately 12 nm from the virion envelope and forms a locally-ordered lattice that interconnects through homodimeric Gc contacts (Figure 4E,F) [154,165,186,187,188]. The N-terminal region of the Gn ectodomain was predicted to locate towards the membrane-distal region of the lattice [165,182]. This hypothesis was confirmed following the integration of a cryoET reconstruction of TULV with the crystal structure of a Gn-Gc heterodimer [154]. This study clarified the handedness of previous lower resolution cryoET maps and unambiguously demonstrated that the N-terminal Gn ectodomain obscures the Gc-resident fusion loops through the formation of extensive protein and glycan contacts with Gc domain II [154]. Additionally, the complex of the Gn-Gc heterodimer also revealed a limited structural role of the Gc N-terminal tail in stabilizing the interactions between Gc domains I and III, in the pre-fusion state [154]. The positioning of the Gc and the nature of Gc-mediated cross-linking of adjacent (Gn-Gc)_4_ spikes was also further verified by integrated X-ray and cryoET analysis of HTNV VLPs in complex with the Fab fragment from a bank vole-derived neutralizing monoclonal antibody (mAb) specific to the Gc glycoprotein [168]. Future work will likely be focused on elucidating the molecular basis for the interaction of the hantaviral (Gn-Gc)_4_ spike complex with host cell surface receptors (Table 1) and how this interaction facilitates uptake of the virus into a host cell.

### 5.4. Nairoviruses (Family Nairoviridae)

Limited structural data currently exists for the nairovirus envelope glycoproteins Gn and Gc. A recent study reported the ultrastructural organization of the Hazara virus (HAZV) envelope glycoproteins to 2.5 nm resolution, revealing that the Gn-Gc assembly displays a putative tetrameric architecture [193]. Due to the low resolution of the reconstruction, it remains unclear which parts represent the Gn and Gc glycoproteins. Given the limited size of the Gn across nairoviral lineages, with respect to the Gc [66], it has been postulated [193] that the membrane distal density may correspond to a portion of the Gc ectodomain, with the Gn being confined to a membrane proximal location. 

Of the nairovirus envelope glycoproteins, only a molecular-level structure of the CCHFV Gn tail has been reported to date using NMR spectroscopy [194]. CCHFV Gn CT (~100-residue) contains dual cysteine/histidine rich motifs (C-X-C-X-H-X-C), which are conserved across many bunyavirus families, including orthobunyaviruses, hantaviruses, and tospoviruses. Furthermore, structural analysis revealed a pair of tightly arranged dual ββα zinc fingers similar to those hantavirus Gn CTs [194]. 

While little is known about the structure of the Gn ectodomain, the structure of the secreted CCHFV GP38 glycoprotein has been recently elucidated and was shown to consist of a three-helix bundle and a β-sandwich (Figure 6B). Interestingly, GP38 has been postulated to share structural features with the ectodomain of the CCHFV Gn glycoprotein due to a gene duplication event [175]. Future high resolution structural studies of the nairovirus Gn and Gc are needed to clarify their mode of assembly and respective functionalities. Additionally, determination of the nairovirus Gc structure will reveal whether the glycoprotein forms the predicted class II fusion glycoprotein architecture observed in hantaviruses, phenuiviruses, alphaviruses and flaviviruses [148].

### 5.5. Tospoviruses (Family Tospoviridae)

The recent structural elucidation of a Gn ectodomain region from TSWV provides the first detailed insights [167] into the glycoprotein architecture of this important group of viruses. TSWV Gn presents structural features similar to the Gn of hanta- and phenuiviruses and the E2 of alphaviruses, consisting of a pincer domain (analogous to the β-ribbon domain), an N-terminal domain A, and a domain B that is inserted between two β-strands of the β-ribbon/pincer domain (Figure 5). Interestingly, the TSWV Gn crystal structure presents a C-terminally positioned domain of a topology similar to that observed in the C-terminal ectodomain regions of hantavirus Gn, alphavirus E2 and flavivirus prmE (Figure 6) [154,170,195]. Distinctively, TSWV Gn contains a reduced version of domain B, which forms a β-hairpin that faces the opposing domain A. Studies of the glycoprotein assemblies found on tospovirus particles indicate the existence of both Gn homodimers and Gn/Gc heterodimers [196] While partial disulfide bond-mediated dimerization of Gn was observed in solution, crystal contacts of the reported TSWV Gn structure may reflect a second dimerization mode through its pincer domain (equivalent of the β-ribbon domain) [167]. 

Similar to other bunyaviruses, the Gc protein of tospoviruses has been predicted to be a class II fusion protein [197] and, based on sequence alignments, most closely resembles that of orthobunyaviruses [162]. High resolution structural characterization of the orthobunyavirus and tospovirus Gc glycoproteins will be necessary to validate this hypothesis. Given the structural distinctiveness of the tospovirus Gn with respect to other bunyavirus Gns [167,196], it seems possible that the tospovirus envelope Gn/Gc assembly may represent another variation on the assembly modes of bunyaviruses. The strong conservation of Gc fusogens, combined with greater structural diversity of the Gn glycoprotein (Figure 5), suggests an instrumental role for the latter protein for dictating the distinct ultrastructural glycoprotein organizations on the viral membrane.

### 5.6. Arenaviruses (Family Arenaviridae) 

Although some cryoET analysis has been performed for the University of Helsinki virus (UHV) (genus *Reptarenavirus*) [198], which demonstrated a trimeric glycoprotein assembly reminiscent to the GP of the Ebola virus [155,199], structural information of the envelope glycoproteins within the *Arenaviridae* family is mostly limited to the GP from the *Mammarenavirus* genus. Each protomer of the highly N-linked glycosylated trimeric mammarenavirus GP is composed of a non-covalently associated heterotrimer consisting of an SSP, GP1, and a transmembrane GP2 [155,200]. The non-covalently associated GP1 is responsible for receptor recognition and has been hypothesized to be shed from the GP following exposure to acidic pH during internalization of the virus into a host cell [155,201,202]. 

Crystal structures of the GP1 have been solved for both New World (NW) and Old World (OW) arenaviruses and shown to be composed of a compact α/β fold (Figure 7C) [203]. Studies of the OW GP1 from LASV, lymphocytic choriomeningitis virus (LCMV), Morogoro virus (MORV), and Loei River virus (LORV) have revealed contrasting conformations when produced alone or in complex with the cognate GP2 [204,205,206,207,208]. While a NW GP1-GP2 structure has yet to be reported, the NW GP1 from Machupo virus (MACV) [209,210], Junín virus (JUNV), and Whitewater Arroyo virus (WWAV), solved alone, and in complex with the TfR1 receptor and Fab fragments of neutralizing mAbs, have revealed only a single conformation, which may resemble the GP2-bound pre-fusion state [207,209,210,211,212,213,214]. The observation that the OW arenavirus GP1 undergoes conformational changes provides a structure-based hypothesis for how it may constitute an immunological decoy following release from the pre-fusion GP complex during infection [202,215,216,217] and is consistent with a study showing that recombinantly-derived NW GP1 is more effective at raising a neutralizing antibody response than OW GP1 [217]. Interestingly, crystallographic analysis of receptor-bound Lujo virus (LUJV) GP1 revealed a structure that contrasts known OW and NW GP1 structures [218], an observation that likely reflects its unique usage of the NRP2 host cell molecule (Table 1) [103]. 

Unlike the other bunyaviruses reviewed above, detailed structural data has been acquired for several arenavirus GP-receptor interactions. X-ray crystallography of a MACV GP1-hTfR1 complex provides a structural basis for recognition of human TfR1 by the GP1 of certain zoonotic clade B and D NW arenaviruses [210,219]. Interestingly, this binding site on TfR1 is distal from that used by the physiological ligands transferrin and hereditary hemochromatosis associated protein [210]. The crystal structure of a LUJV GP1-NRP2 complex revealed a metal ion-dependent mechanism of recognition that may involve the full trimeric spike during native binding [218]. Finally, although limited information exists for how most OW arenaviruses and clade C NW arenaviruses interact with the C-type lectin, DC-SIGN, or the O-mannose glycans presented on α-DG [92,93,101,220], functional studies have identified residues on LASV GP1 that are important in modulating the pH-dependent recognition of LAMP1 [205,206] and a cryoET-derived reconstruction of acidified LASV VLPs in the presence of recombinantly-derived LAMP1 supports this interaction taking place at a membrane distal region of the molecule [155].

CryoET ultrastructure analysis of mammarenavirus GPs, as presented on the virion membrane, has been limited to LASV and has revealed that the GP forms a tripodal organization that extends approximately 9 nm from the virus surface and contrasts the single stem assembly of the UHV reptarenavirus [198]. The low resolution (~1.4 nm) of the reconstruction of LASV GP was largely in agreement with the crystal structure of a trimeric LASV GP1-GP2 complex bound to the Fab fragment of a neutralizing mAb, termed 37.7H [156]. Interestingly, several mAbs (including mAb 37.7H) have been shown to target a site, termed ‘GPC-B’, and to specifically bind a quaternary epitope thereby cross-linking the GP1-GP2 protomers [156,201,221]. These structural data provide first molecular-level insights into the trimeric GP1-GP2 architecture (Figure 4G,H). 

Upon endocytic uptake and detachment of GP1, GP2 is responsible for catalyzing the merger of viral and target membranes in a pH-dependent process. In contrast to other structurally characterized bunyavirus fusion glycoproteins reviewed above, the mammarenavirus GP2 has been shown to be a class I fusion protein [222]. Other class I fusion proteins have been observed in paramyxoviruses, coronaviruses, HIV, and influenza viruses [203,223]. Crystal structures of the GP2 when bound to the GP1 glycoprotein have revealed a GP1-stabilized pre-fusion conformation (Figure 7C), while post-fusion structures of GP2 fragments have shown that the glycoprotein forms a trimeric coiled-coil (Figure 7D) [156,176,201,204,221,223,224,225]. The NMR structure of the GP2 cytoplasmic tail has also been determined and shown to comprise a zinc binding domain, which interacts with the SSP [226].

## 6. Concluding Remarks

Bunyaviruses include several important human, animal and plant pathogens. Elucidation of the biological structure and function of bunyaviral glycoproteins is essential for the rational development of vaccines, drugs and other preventive strategies. Our improving understanding of bunyavirus glycoproteins has enhanced our appreciation of the pathobiological diversity within this important group of pathogens.

However, there remain many questions and challenges concerning the function and structure of bunyavirus glycoproteins. Indeed, much remains to be elucidated on the process of bunyavirus glycoprotein folding and biosynthesis, their diverse assemblies and host-interactions, and how they interact and recruit the N protein during virus assembly. Furthermore, it remains poorly understood how virus pathogenesis differs between human and animal pathogens. We anticipate that clarification of these fundamental elements of bunyavirus biology will further rely on the utilization of cutting-edge technologies and approaches. For example, haploid screening approaches have been essential for the identification of several bunyavirus host cell receptors [94,103,111,123], and may continue to serve as key method for the identification of novel bunyavirus receptors [227]. Similarly, while integrated cryoEM/cryoET and X-ray studies have provided numerous insights into the glycoprotein structure of phenuiviruses, hantaviruses, nairoviruses, and orthobunyaviruses, the low level of glycoprotein sequence conservation across the order suggests that there is still much to learn about bunyavirus glycoprotein assembly and functionality. Indeed, this paucity of structural knowledge both hinders our understanding of how bunyaviruses utilize their glycoproteins to interact with specific host cell types and limits our ability to rationally target these important pathogens. 

Since the recovery of BUNV by reverse genetics from cDNA clones of viral genomes [228], numerous viruses from different families in the order *Bunyavirales* have successfully been rescued, including LCMV, JUNV, LASV, LUJV, Pichinde virus (PICV) (*Arenaviridae*) [229,230,231,232,233,234]; BUNV, LACV and AKAV, SBV, OROV, Cache Valley fever virus (CVV) and Kari virus (KRIV) (*Peribunyaviridae*) [228,235,236,237,238,239]; RVFV, UUKV and SFSTV (*Phenuiviridae*) [240,241,242]; CCHFV and Hazara virus (HAZV) (*Nairoviridae*) [243,244], and TSWV (*Tospoviridae*) [245]. The broader availability of reverse genetics techniques will empower studies of bunyavirus pathobiology and aid the development of preventive strategies (e.g., vaccine and antiviral development) that may be used to protect against infection. 

In summary, bunyaviruses constitute a growing order of biomedically and economically impactful pathogens. Continuation of the already successful efforts to characterize these viruses will not only enhance our appreciation of the seemingly limitless genomic, structural, and functional diversity within the order, but also enhance our preparedness for their future emergence. 

## Figures and Tables

**Table 2:** Receptor-mediated endocytosis pathways used by bunyaviruses

Endocytosis Pathway	Viruses
Clathrin-mediated (CME)	OROV [132], LACV [133] (*Orthobunyavirus*) SFTSV [134,135] (*Bandavirus*) HTNV, SEOV [136]; ANDV [137] (*Orthohantavirus*) CCHFV (*Orthonairovirus*) [138,139] JUNV [140], PICV and LASV [141] (*Mammarenavirus*)
Caveolin-1-mediated (CavME)	RVFV (*Phlebovirus*) [142] ANDV [137]
Clathrin and caveolin independent	AKAV (*Orthobunyavirus*) [143] UUK (*Phlebovirus*) [144] LCMV (*Mammarenavirus*) [145,146]
Macropinocytosis-like	ANDV (*Orthohantavirus*) [137]
